# Ethical Challenges Involved in COVID-19 Vaccine Mandates for Children: A Systematic Review

**DOI:** 10.3390/vaccines11030601

**Published:** 2023-03-06

**Authors:** Ghiath Alahmad

**Affiliations:** King Abdullah International Medical Research Center (KAIMRC), King Saud Bin Abdulaziz University for Health Sciences, Riyadh 11481, Saudi Arabia; ghiathalahmad@hotmail.com; Tel.: +966-539-459-000

**Keywords:** COVID-19 vaccine, mandates, vaccine hesitancy, vaccines, autonomy, beneficence, non-maleficence, coercion, organ transplantation

## Abstract

The new COVID-19 pandemic has affected day-to-day life, creating various ethical dilemmas. COVID-19 vaccination is seen as an effective way to halt the pandemic. Ethical challenges can arise when the vaccines are mandated for all ages, but more so when mandated for children. This systematic review discusses the pros and cons of the COVID-19 vaccine mandate for children. The primary objective of this study is to summarize exclusively the various ethical conflicts, impacts, and requirements that arise as a result of the COVID-19 vaccine mandate laws on children. The secondary objective is to analyze the reasons for parents refusing to allow their children to be given the COVID-19 vaccine sand the effective strategies to increase vaccine uptake among children. The study involved a systematic review, identification of relevant literature and reviews following the PRISMA-ScR recommendations. The keywords ‘COVID-19 vaccine mandates on children’ were used to mine the literature from PubMed and WHO COVID-19 Research Database. Limitations placed on the original searches were: English language, humans, ethics, and children. Out of 529 studies, only 13 satisfied the selection criteria. The sample included studies with a wide, diverse range of methods, settings, research, authors, and journals. COVID-19 vaccine mandates on children need to be scrutinized. Implementing the COVID-19 vaccination drive in a scientific way is acceptable. As children are the fastest-growing population and have the highest life expectancy, it is important to take into account that the vaccines do not disturb their growth and development.

## 1. Introduction

The first case of COVID-19 was reported in late December 2019 [1] in Wuhan, China [2]. Soon after, it became a global pandemic with 290,000,000 confirmed cases and 5,446,753 deaths over the past two years all over the world [3]. Many countries commenced developing vaccine soon after the pandemic [4] with the aim of preventing infection-associated morbidity and mortality [5]. For a vaccine to be developed and made available, it would normally require a minimum duration of at least 12–18 months. However, considering the severity of the situation and the necessity to decrease the risk of COVID-19 transmission and death [3,4], the US Food and Drug Administration (FDA) has granted emergency use authorization for treatment with the Pfizer/BioNTech and Moderna COVID-19 vaccines [6]. Most countries have promoted a vaccination drive and some have mandated adult vaccination. They are now focusing on vaccinating children aged 12 to 15 years [7] with the newly formulated vaccine for children produced by Pfizer-BioNTech and Moderna [5]. Vaccinating children aged 5 to 11 years is also being considered [7].

SARS-CoV-2 infection poses a serious risk to the elderly population (over 65 years of age) and those with comorbidities, such as diabetes, hypertension, cardiovascular diseases, malignancy, and obesity. However, children are at a lower risk of being infected with SARS-CoV-2 [5]. According to World Health Organization (WHO) reports, the mortality rate in children below the age of 14 is 0.1% [8]. The lack of enough data regarding the effectiveness of vaccines [5] and the short- and long-term side effects of vaccination in children [9] has led to significant hesitancy among parents about vaccinating their children [8].

Ethical challenges can arise when vaccines are mandated for all ages, but they increase significantly when mandated for children. While relatively little was known about the SARS-CoV-2 virus, and in a climate of urgency for vaccine development, the FDA allowed accelerated development of new vaccines, and Moderna eliminated the animal trials proceeding straight to stage one trials with no knowledge of the long-term effects of the vaccine [4]. This may be the reason that vaccination coverage has declined in many countries [8]. There is also a counterargument stating that voluntarily unvaccinated people are not fulfilling their obligations and could be causing harm to others, especially to the susceptible elderly and immunocompromised population [10]. The aim of this study is to discuss the pros and cons of the COVID-19 vaccine mandate for children. The primary objective of this study is to exclusively summarize the various ethical conflicts, impacts, and requirements that arise as a result of the COVID-19 vaccine mandate laws on children. The secondary objective is to analyze the reasons for parental refusal of COVID-19 vaccines and the effective strategies to increase vaccine uptake among children.

## 2. Materials and Methods

### 2.1. Search Criteria

The study involved a systematic review. A literature search method developed to identify relevant literature was applied following the PRISMA-ScR (Preferred Reporting Item for Systematic Reviews and Meta-analyzes for scoping review) recommendations. The keywords ‘COVID-19 vaccine mandates on children’ were used to mine the literature from the databases PubMed and WHO COVID-19 Research Database. Limitations placed on the original searches were: English language, human, ethics, and children, from 2019 until 31 December 2022.

### 2.2. Inclusion Criteria

The articles were refined to select the ones that met the inclusion criteria. The inclusion criteria set were ethical issues with COVID-19 vaccination mandates for children. The exclusion criteria set were: (1) not about ethics, (2) not about COVID, (3) not about children, (4) not about vaccination, (5) editorial, viewpoints, commentary, or case studies.

## 3. Results

**Figure 1:**
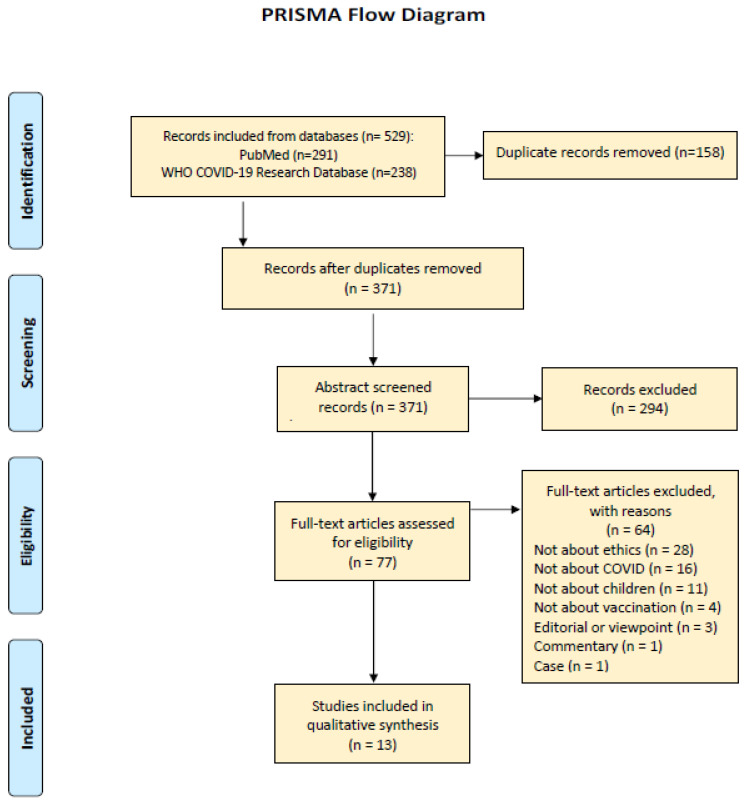
Showing the data screening and extraction with PRISMA.

The search strategy retrieved 529 articles. After removing duplicates, 371 articles remained for title/abstract screening, and 77 full-text reports were assessed for eligibility. Based on the application of the search criteria, 13 articles that met those criteria were refined from online databases. The references and reasons for excluded studies are available in (Figure 1).

We have reviewed various journals and tried to summarize the ethical challenges posed by mandating or denying vaccination for children. The risk-benefit ratio of the pediatric COVID-19 vaccine mandate is examined. Here, we do not take a stand on vaccination but have analyzed both sides of an important issue. We have presented literature that has focused solely on the ethical challenges of COVID-19 vaccine mandates for children. The demographics of the studies are given in (Table 1).

### Characteristics of the Included Studies

The selected articles expressed opinions both in favor of and against the COVID-19 vaccine mandate for children. The articles selected had a wide range of focus in terms of study, method, place of research, results, and conclusions. The articles discussed coercion, autonomy, beneficence, non-maleficence, parental hesitation, vaccination refusal, organ stewardship, and public and governmental policies regarding the COVID-19 vaccine mandate. Ten of the articles were literature reviews, two of which were argumentative reviews. Two articles were commentaries, and one used a cross-sectional descriptive study. The primary and secondary ethical problems addressed in each article are summarized in (Table 2).

Few studies have focused on the COVID-19 vaccine mandate [4,5,9,11,12]. Among these, [5] discussed implementing the COVID-19 vaccine mandate in a scientific way, and [12] discussed the political and functional needs of COVID-19 vaccine mandates. The other articles discussed the consequences of COVID-19 vaccine refusal and methods to be followed to implement the vaccine mandate. In [3,10,17], the authors discussed COVID-19 vaccine mandates in organ transplantation, organ stewardship, and pros and cons of COVID-19 vaccine mandates for transplantation candidates. In [7,14], the authors spoke out against COVID-19 vaccine mandates, citing unknown side effects. In [8], the author discussed the parents’ attitude towards the COVID-19 vaccine mandate, and in [15], they discussed parental informed consent.

Each article addressed the various ethical issues and challenges posed by the COVID-19 vaccine mandate for children. When children are less susceptible to the COVID-19 infection, mandates are regarded as coercive [4,7]. Safety, efficacy, and unknown side effects were the main reasons for parental hesitancy [8]. However, vaccination is the most effective way to reach herd immunity [9]. The COVID-19 vaccine might endanger the lives of pediatric transplant candidates [3,17]. In this systematic review, we have analyzed and summarized the ethical conflicts and impacts of COVID-19 vaccine mandate on children, the reasons for parental hesitancy, why the COVID-19 vaccine is important for children, and the effective strategies to be handled to reduce parental hesitancy and improve vaccine coverage.

## 4. Discussion

The main themes that emerged from the included studies are listed in (Table 3).

### 4.1. Vaccine Mandates in The Pre-COVID-19 Era

The vaccine mandate has always been a highly debated issue [18], even before the outbreak of COVID-19. Ethical challenges regarding vaccine mandates are a long-standing issue, especially when they are introduced to children. A number of parents have expressed concerns about the safety of vaccines and questioned the need for them. Conspiracy theories circulating on the internet, alternative medicine concept, and civil liberties all contributed to the anti-vaccine ideology among parents even before the COVID-19 outbreak [19]. Certain religious beliefs, such as “vaccination is against the Word of God”, and preaching, such as “vaccines are sinful practices”, have motivated the anti-vaccination attitude. Apart from theological factors that oppose vaccinations, political and legal factors also affect vaccine acceptance [20]. Evidence of hesitancy to use vaccines and outright refusal has been recorded in the past, resulting in serious consequences. The conspiracy theories linking MMR vaccines to autism resulted in declining MMR vaccination rates, which declined from 92% in 1996 to 61% in 2003 in the UK, eventually leading to a measles outbreak in many western countries and causing several deaths [20]. Mandatory vaccination programs have existed in the past, with many nations passing vaccination regulations. This comprises many countries that have made vaccination records a requirement for enrolling in school in order to increase immunization rates. In Uganda, parents who do not vaccinate their children risk receiving a harsh penalty of up to six months in jail [21].

### 4.2. COVID-19 Vaccine Mandate and Impacts

Although children are usually the least affected by COVID-19 infection and have lower mortality rates (0.0016%), the multisystem inflammatory syndrome in children (MIS-C) is a common aftereffect of COVID-19 infection in children and is of great concern [5,14]. SARS-CoV-2 viruses are continuously mutating, and the new variants such as Delta and Omicron have affected children severely. The latter has increased the risk of mortality in children [5]. The strict lockdown measures have refrained children from social activities and going to school, causing severe psychological impact. Vaccinating children is an alternative effective way to handle the pandemic and its effects on society [5].

Although most children are asymptomatic or less severely affected by COVID-19 infections, they may still act as a reservoir for the infection and transmit it. It is reported that COVID-19 vaccines produce a stronger and more durable antibody response in children [5]. The important consideration in mandating the COVID-19 vaccine is the economic burden and accessibility to vaccines [5]. There are various unanswered ethical questions that need to be considered before mandating vaccination. The safety and efficacy of the vaccine on children, children’s susceptibility to the infection, children’s role in the transmission of the disease, and the expected benefits are unknown. With so many unanswered questions, mandating vaccination on children may be considered an injustice [14]. Vaccine mandates, though they can be justifiable in line health ethics, are considered against traditional clinical ethics [16]. These ethical dilemmas challenge ideal vaccine coverage. Children who have recovered from COVID-19 have gained natural immunity against the disease, and Norway no longer recommends vaccinating children aged 12–15 who have recovered from COVID-19 [7].

#### 4.2.1. Coercion

In contrast to many vaccine-preventable diseases, the risk of serious illness from COVID-19 infection among healthy children is much lower. Hospitalization and mortality cases are rare in children [7]. In such cases, vaccine mandates are coercive. A few nations, such as the US [14], have imposed laws such as: unvaccinated children are forbidden from attending school [5,16]. In a few other countries, financial assistance payments are withheld if families refuse to vaccinate their children. A few other nations, such as France, Italy, and Australia [16], also consider COVID-19 vaccine refusal a crime and consider imprisonment [5,14] or imposing fines [16] on parents who do not comply with the law. Those who refuse to comply with the vaccine mandate may face penalties from the government. These penalties may range from attending educational programs to financial penalties, or sometimes they can be as severe as imprisonment [14]. Coercion poses a threat to the principles of autonomy, liberty, and freedom [14]. Sometimes children may consent to COVID-19 vaccination due to their altruistic nature [7].

#### 4.2.2. Autonomy 

Children usually have less autonomy; they are considered to lack the capacity for decision-making [18] and depend on their parents or elders to make decisions [4]. In many medical care systems, autonomy can be reduced due to the risk to others and self-harm caused by negligence of care [4,10,11]. The mandate for the COVID-19 vaccine has created a quandary about which to prioritize; public commitment and responsibility to prevent outbreak and transmission or individual autonomy and freedom [16]. The mandates override parental autonomy [7]. According to national and international conventions, children should be involved in the decision-making process in medical treatment. A process of co-decision or shared decision-making results in the formation of a triple therapeutic alliance involving the doctor, parent, and child [15].

#### 4.2.3. Beneficence and Non-Maleficence

Although most of the infected children are asymptomatic or mildly ill, the long-term effects of the disease are still unknown [4]. The current technological advancements have increased the ability to produce vaccines that are highly effective with minimal side effects [4]. Even though rare, children can become seriously ill due to COVID-19, and the vaccine against it has proven to provide some benefits for children [5]. However, the safety, efficacy, and long-term effects of the vaccine remain unknown. It is possible that any harm incurred due to vaccination will be revealed to the public only after millions of children have already been vaccinated [7]. Recently, it was identified that AstraZeneca vaccines caused blood clotting events in older age groups [7], and COVID-19 mRNA vaccines resulted in myocarditis in young men [22] and adolescents [23]. These risks came to light only after millions of people were vaccinated, and a few deaths were reported [7]. Countries such as Sweden, Denmark, France, and Germany have halted the use of Moderna’s COVID-19 vaccine due to reports on the cardiovascular side effects [7]. It is argued that the COVID-19 vaccines are futile and provide only short-term immunity. There are also chances of the viruses evolving due to immune escape mutations [5]. The Oxford vaccine is the only one that has been tested on children aged 5 to 12, and phase III trials are currently underway. In such a scenario, the safety of the vaccine remains unknown. Although the expected efficacy of the COVID-19 vaccine is high, it is still unproven [14].

#### 4.2.4. Justice

Children are less susceptible to COVID-19; the disease severity in children is mild, and the potential benefits of the COVID-19 vaccine are low, so mandating vaccines is untenable [7,9]. If vaccines are mandated, children should be given the opportunity to receive them [5]. There is a need for fairness in the laws that mandate vaccination. When mandatory vaccination laws penalize refusal, steps should be taken to compensate the child, family, and community for vaccine-related adverse events [5].

### 4.3. Parental Hesitancy and Refusal

Parental hesitancy is a complex issue that relates to concerns about vaccine efficacy, effectiveness [3], and long-term side effects [5,8]. The COVID-19 vaccine mandate has obliged parents to make decisions about their children being vaccinated [5]. The WHO has listed vaccine hesitancy as one of the ten major threats to global health that commonly occur in high-, middle-, and low-income countries (LMICs) [12]. There are various reasons for parental hesitancy.

#### 4.3.1. Religious Beliefs

Evidence of vaccine hesitancy because of religious belief has been recorded in the past. Though the impact of religious beliefs on vaccine hesitancy has been considerably reduced in the present world, it still persists. Religious beliefs and vaccine hesitancy differ and change across different groups of people. There was no link found between religiosity or practice of a major faith tradition and parental vaccine hesitancy among Latino Christian mothers in America, whereas vaccine hesitancy was common among Evangelical Christians in America [24]. In a survey conducted among 27 European countries, vaccine hesitancy was exhibited by 18% of the total participants (n = 42,583) who prayed daily and 11.9% of the participants who never prayed, showing that COVID-19 vaccine acceptance was higher in the people who never prayed [25]. In Bangladesh, COVID-19 vaccine hesitancy among parents was more prevalent among the Muslim population than the non-Muslim population [26].

#### 4.3.2. Safety Concerns

A parental refusal to receive the COVID-19 vaccine does not mean that the parents are denying their duty or neglecting the child’s health, but it is mostly the outcome of hesitation due to the unknown harm that might be incurred due to the COVID-19 vaccines [7]. A survey by Mohan et al. (2022) states that though parents generally have a positive attitude and acceptance about vaccinating their child, the majority of them are concerned about the unknown side effects of vaccination, and people who either themselves or family members were not diagnosed with COVID-19 considered naturopathy a better option [8]. These hesitations have resulted in a large number of vaccines being wasted all over the world [17,27]. It has been reported that countries such as China, Italy, England, the United States, Canada, and Israel have a high rate of parental hesitation for vaccinating their children [5].

#### 4.3.3. Informed Consent

The informed consent of parents has created various issues in its practical application. Certain legal systems require the consent of both parents, regardless of whether they are married, cohabiting, or divorced. Sometimes the parents’ decision is against the child’s wishes. In such cases, it is important to consider what is in the best interest of the child. The legal system can also intervene and appoint specialized judges to investigate the case and provide verdicts [15].

### 4.4. COVID-19 Vaccine Mandate and Impact on Pediatric Organ Transplantation

The American Society of Transplantation, the International Society for Heart and Lung Transplantation, and the American Society of Transplant Surgeons have proposed a joint statement on 13 August 2021, strongly recommending the COVID-19 vaccination in transplant candidates, recipients, and other household caregivers who are in close contact with the recipient, thereby emphasizing organ stewardship [10,13]. Organs are limited resources that need to be rationed and considered for the best use by maximizing the significant medical benefit to the recipient. The organ stewardship [12] and gatekeeping approaches are critical in ensuring that the appropriate candidate receives the greatest benefit and survival chance from transplantation [13]. Transplantation increases the risk of SARS-CoV-2 infection due to weakened T-cell-mediated immunity, and vaccination is the only option to develop immunity. A few kidney transplantation centers [3] and heart and lung transplantation centers [13] have mandated COVID-19 vaccines prior to transplantation. The vaccination is mandated in the pediatric age group of 5 years and above, except for children whose parents refuse vaccines for their children. It is reported that 5% of the kidney transplant patients were infected with vaccine-preventable diseases, and the risk was four times higher among the unvaccinated transplant recipients [3]. When a parent refuses to vaccinate their child prior to transplantation, they may be exposing to greater risks of infection not only for their own children but also to other pediatric transplant patients, as transmission in the waiting room is possible [3].

Parents who refuse vaccines for themselves put their children at risk, especially in transplant cases [10]. During the pre-transplantation stage, the children are expected to maintain a certain level of health in order to be eligible for the surgery [10]. Any COVID-19 infection during this phase might also result in the postponement of the surgery, which might cause a severe risk to life. Because of the immunosuppressant, the post-transplant stage increases the risk of COVID-19 [10]. Again, any infection during this stage might create an ethical dilemma between treating the infection or saving the transplanted organ and avoiding graft rejection. Voluntarily unvaccinated parents are neglectful of these complications [10].

A recent report states that among unvaccinated pediatric kidney transplant recipients, the risk of SARS-CoV-2 infection is only mild, and none of the cases resulted in allograft loss or death, although the chronic outcomes are still unknown [3]. While the risk of SARS-CoV-2 infection and mortality post-transplantation is higher due to immunosuppression, this has not been demonstrated or proven in children [3]. The vaccine mandate arguments in pediatric transplant groups are weaker [10], and the evidence supporting the mandate of the COVID-19 vaccine among pediatric kidney transplant patients is limited [3]. Though COVID-19 vaccination prior to transplant is preferable for the recipient and their family members, the magnitude of benefit is unknown [3,10]. In such a scenario, the vaccine mandate focuses on the ethical principles of beneficence by maximizing the benefit of organ transplantation and justice towards the donated organs, which are scarce resources. A vaccine mandate for transplantation patients jeopardizes the principles of autonomy and non-maleficence because the benefits and risks are unknown [3].

In the case where the risk of infection in pediatric transplant recipients is very low, the vaccine mandate may not be justifiable [3,13]. Though refusal of vaccination may incur harm to the transplantation candidate, the harm incurred due to the un-listing of candidates for organ transplantation who or whose family members refuse to get vaccinated is greater [13]. Vaccine mandates are coercive, denying the child the opportunity to receive transplantation surgery, posing a threat to the child’s life, and posing a higher risk of mortality than COVID-19 itself [3,13]. It would be inappropriate and unethical if the vaccine mandated inequity among the children who are listed for transplantation [3].

The three-dose vaccine coverage for pediatric liver transplant (n = 563) and hematopoietic stem cell transplant (n = 122) recipients was 0.9% and 4.9%, respectively. Fear of vaccine-induced adverse events and doubts about efficacy were the main reasons for vaccine hesitancy. Most children infected with Omicron have mild or no symptoms, and the infection was transmitted by family members who were hesitant to vaccinate their children. It would be effective to encourage the parents and other family members to get vaccinated, possibly reducing the chance of infecting the children [28]. In an observational study reported by the Johns Hopkins University of Medicine, it was reported that after receiving both doses of the vaccine, no graft rejection or allergic reactions were reported by pediatric transplant patients [29]. Such valuable information has to be conveyed to the parents who are hesitant about vaccinating their children.

### 4.5. Public Policies and Impacts

There are many ongoing research studies about COVID-19, and a huge number of unknowns need to be understood, and much uncertainty needs to be clarified. However, with this level of uncertainty, it is also an obligation of the government to make public policies in the best interest of the citizens, with the known information. Sometimes policies amended in the best interests of the public can impact autonomy, utilitarianism, and justice [4]. The pressure to license the vaccine quickly and to make it available for public use may elicit social fear and hesitancy in vaccine uptake. Shortening the vaccine development process or skipping stages in clinical trials may lead to safety problems, where the undiscovered and unexpected risks need to be elucidated [4]. Such events have happened in the past with rotavirus and flu vaccines, where people reported intestinal problem with the former and narcolepsy with the latter, eventually leading to the retraction of vaccines from the market [4]. The law of mandating vaccines can be considered ethical if it does not affect the ethical principles or disturb personal right and freedom and does not pose any harm [5]. A vaccine mandate policy might increase public polarization [12] and might create a dichotomy between anti-vaccinators, who do not comply with the law and cause a serious ethical violation, and health scientists and physicians who neglect the ethical issues [16].

Mandates imply coercion [9], which might agitate the reaction towards COVID-19 vaccination and increase anti-vaccination attitudes among the public. Certain communities believed that COVID-19 was fictitious and only a conspiracy theory, and they started protesting against the mandatory rules on masks and homestay [12]. The protestors had very little confidence in the public healthcare systems. This protest became even louder against COVID-19 vaccine mandates. Mandates may undermine public health ethics and integrity [12], whereas alternative policies such as quasi-mandates, incentivization, and facilitation might prove to be more appropriate in encouraging vaccination [9]. When the public’s confidence about getting vaccinated remains low, the government might be pushed to impose vaccine mandate laws [16].

#### Incentives

Though incentives have been found effective in a few programs, such as adolescent diabetes self-care programs [30], they also raise various ethical concerns such as being exploitative or creating undue inducements [14]. Serbia, in May 2021, was the first nation to incentivize vaccination by paying USD 30. Hong Kong and the United States have also begun to support COVID-19 vaccination incentives [31]. In a survey among US adolescents, only 7% of the total participants (n = 1125) reported that incentives would influence them to uptake the COVID-19 vaccine, with most of them expressing concerns about the safety and effectiveness of the vaccine [32]. Though monetary incentives are often controversial, it is an undeniable fact that they have substantially improved vaccine uptake rates in many countries (4.2% in Sweden) [33]. In Germany, about 19% of the participants opted to get vaccinated when a large sum of EUR 10,000 was offered. On the other hand, monetary incentives decreased the confidence about vaccination among others [34], whereas in Ohio, where the Vax-a-Million lottery seemingly decreased vaccine acceptance by 29.7%, and when USD 100 incentives were offered, 6.8% were motivated to take vaccin, whereas 17.4% reported a decreased likelihood of COVID-19 vaccination [35]. In Israel, incentives did not encourage parents to vaccinate their children [36]. Incentives have effectively spurred vaccine uptake in low- and middle-income countries [37].

### 4.6. COVID-19 Vaccination Status in LMIC

Vaccine acceptance is higher in LMICs (80.3%) than in upper-middle (30.4%) and high-income countries (64.6%) [38]. However, only 17.4% of the population in LMICs had received their first dose, showcasing the limited access and availability of the COVID-19 vaccine [39]. According to research, 49% of LMIC parents agreed to vaccinate their children and believed that COVID-19 vaccines would be effective in containing the pandemic [40]. The worldwide parental acceptance rate for COVID-19 vaccines is 58% [41]. Communication and policies that provide credible information about vaccination [42], parents’ knowledge and trust about vaccine, cost, accessibility, and government incentives were the factors that encouraged parents to vaccinate their children [41]. Vaccine acceptance is not directly proportional to a nation’s income distribution, but rather to an individual’s income and educational qualifications, belief in the vaccine’s ability and safety, and trust in vaccine science [39]. On average, global parental vaccine hesitancy has decreased by 5.9%, but increased in nations such as the UK (10.9%), Russia (10.2%), South Korea (49.8%), South Africa (40.4%), Poland (2.4%), Ghana (12.1%), Germany (8.7%), and Brazil (56.3%), whereas it has decreased in many LMICs [38].

### 4.7. Necessity of COVID-19 Vaccine Mandates

As aforementioned, a few nations have already witnessed the consequences of vaccine refusal in the past and have now strongly enforced laws to mandate COVID-19 vaccination [43]. Voluntarily unvaccinated people increase the chance of SARS-CoV-2 mutation and transmission, thereby increasing the risk of infection among people who cannot be vaccinated for medical reasons, children who have severe allergic reactions to vaccines, and immunocompromised elders. Thus, when a voluntarily unvaccinated person contracts COVID-19, they ultimately use healthcare resources that were intended for another ill person, which could have been avoided if vaccinated. Thus, in such a scenario, it is imperative to balance the allocation of medical care and lessen the priority given to those who have not complied with the vaccine mandate rule. This might forcefully encourage more people to get vaccinated [10]. COVID-19 vaccines are considered safe and effective in preventing the acquisition and transmission of the virus, thereby preventing hospitalization and death. Despite the availability of safe vaccines, many children remain unvaccinated [3]. Though the risk of healthy children being infected with COVID-19 is very minimal, they can be a carrier and transmitter of the disease to other vulnerable children, children with comorbidity, or to other adults such as teachers in a classroom environment, or to elders who are immunocompromised [4].

### 4.8. Strategies to Improve COVID-19 Vaccine Uptake in Children

In the United States, approximately 68.8% of parents expressed reluctance to vaccinate their children under the age of five. Most parents believed that children were not susceptible to COVID-19 and that vaccines were not effective against the new variants. Improved public health messaging, targeted messaging [44], and governmental campaigns involving pediatricians could be effective strategies to increase awareness among parents and reduce parental hesitancy [45].

#### 4.8.1. Vaccination Campaigns

Vaccination campaigns are considered an effective strategy to eliminate misunderstandings about the vaccine and improve vaccine uptake. Almost every country is engaged in rigorous vaccination campaigns that focus on improving COVID-19 vaccination awareness. In the Republic of Cyprus, where the vaccine was not mandated, an intense COVID-19 vaccination awareness campaign successfully resulted in 60% of the adults being fully vaccinated [46]. Most of the parental hesitancy is due to concerns regarding the safety and efficacy of the vaccine. A well-planned campaign to raise awareness about the benefits of vaccination and to eliminate misinformation, concerns, and fears that reach reluctant parents would successfully increase pediatric vaccine uptake [47].

#### 4.8.2. Parent-Physician Interactions

Face-to-face interactions about the importance of vaccination with people who lack awareness were found to be an effective strategy to reduce vaccine hesitancy [43]. Effective communication and sharing information can increase confidence in parents about vaccinating their children.

#### 4.8.3. Role of Media

Physicians and the media play a great role in increasing parents’ trust [5]. The mass media plays a crucial role in creating public awareness and should deliver impeccable information to the public. In a survey by Horiuchi et al. [48], it was stated that about 38% of the population trusted mass media as a reliable source of information [49].

#### 4.8.4. School Mandates

Many countries have mandated the COVID-19 vaccination to be administered in schools. School mandates are considered effective for reducing the transmission of disease [4]. Vaccine mandates will increase the vaccination rate and boost herd immunity, which can be achieved only when 70–80% of the population is vaccinated [5]. The vaccines are found to be more effective in children than in elders or immunocompromised adults; hence, vaccinating children would be an effective way of achieving herd immunity. It is also argued that using children for the benefit of the community may be unethical and immoral [5]. Nevertheless, the limitation depends on the perfect balance between the risk-benefit ratio of the vaccines [4].

The COVID-19 pandemic has always posed new challenges to the public, the government, the healthcare sector, and scientists. Mandates have always been enforced, right from the start of the pandemic. Mask requirements, social distancing, sanitizing, strict lockdown measures, and restrictions on public gatherings have always been in place [49]. These mandates, though necessary to keep the transmission in check, were not the most effective way to halt the pandemic. Given that children are less susceptible to COVID-19 infection, a vaccine mandate for them was deemed unjust and an infringement on their autonomy [7,9]. The COVID-19 vaccine is considered the most efficient way to combat the virus and halt the pandemic, and vaccinating children is considered the effective way to attain herd immunity [15]. Though few welcome the COVID-19 vaccine mandate rule, it has also evoked counter-reactions among the public. Vaccine mandates are considered coercive and restrict individual or parental autonomy, liberty, and freedom for the benefit of the public or the children’s own good [15]. When the COVID-19 vaccine was made mandatory for children, parental skepticism was high. The mandate was considered coercive, and the main reason for parental hesitancy was the concern regarding the safety and efficacy of the vaccine [47].

## 5. Conclusions

In general, vaccine refusals increase the chance of outbreaks of more vaccine-preventable diseases worldwide, as evidenced by past occurrences. A COVID-19 vaccine mandate can be considered ethical if the risk is low and access is readily available. It is the obligation of the transplantation team to listen to children’s and parents’ concerns about vaccination, educate them, and clear their doubts and fears, eventually persuading them to undergo the vaccination. Implementing the program in a scientific way that does not pose a financial burden to families is acceptable, and in such cases, a financial incentive can be considered a better option. However, in a massive vaccination drive, it may not be practically possible, particularly for LMICs, considering the financial crises caused by COVID-19. As children are the fastest-growing population and have the highest life expectancy, it is important to take into account that the vaccines do not disturb their growth and development. COVID-19 vaccine mandates need to be scrutinized. It is the obligation of the government to improve the vaccination drive not just by enforcing the laws but also by taking necessary measures to improve the public’s understanding and knowledge about COVID-19 vaccination and eradicate public fears regarding COVID-19 vaccination before conducting a mass vaccination drive.

## Figures and Tables

**Table 1:** Demographics of included articles.

Authors	Title of Article	Date of Publishing	Research Methods	Sample	Place of Research
Archard et al. [9]	Compulsory childhood Vaccination: human rights, Solidarity, and best Interests	2021	Commentary		UK
Assadi et al. [5]	COVID-19 vaccination in children as a global dilemma through an ethical lens: A retrospective review	2021	Review		Iran
Carrion and Bramstedt [10]	Exploring the ethical complexity of pediatric organ transplant candidates and COVID-19 vaccination: Tensions between autonomy and beneficence, children and parents	2022	Review		UAE and Australia
Iserson [11]	Ethics, Personal Responsibility and the Pandemic: A New Triage Paradigm	2021	Review		Arizona
Kraaijeveld et al. [7]	Against COVID-19 vaccination of healthy children	2022	Review		Netherlands, USA, UK
MacDonald [12]	Vaccines, Politics and Mandates: Can We See the Forest for the Trees?	2022	Commentary		Canada
Mohan et al. [8]	Acceptance and attitude of parents regarding COVID-19 vaccine for children: a cross-sectional study	2022	Cross-sectional descriptive study	204 parents of children aged between 2–15 years	India
Reiss and Caplan [4]	Considerations in mandating a new COVID-19 vaccine in the USA for children and adults	2020	Review		USA
Ross and Opel [13]	The case against COVID-19 vaccine mandates in pediatric solid organ transplantation	2022	Argumentative Review		USA
Savulescu [14]	Global Ethical Considerations Regarding Mandatory Vaccination in Children	2021	Review		
Scendoni et al. [15]	Legal and ethical issues around COVID-19 vaccination consent in Italian children from 12 years of age	2021	Review		Italy
Wightman et al. [3]	Considering a COVID-19 vaccine mandate for pediatric kidney transplant candidates	2022	Review		USA, Canada
Williamson [16]	The ethical impact of mandating childhood vaccination: The importance of the clinical encounter	2021	Argumentative Review		USA

**Table 2:** Primary and Secondary Ethical issues focused in included studies.

Authors	Main Ethical Problem(s)	Secondary Ethical Problems/ Ethical Applications/Outcomes/Challenges/Consequences	Limitations/Outcomes
Archard et al. [9]	Mandating vaccination	Coercion Protection of children from infectious diseases Consequences faced by parents due to refusal	Decrease in voluntary vaccination results in decrease in herd immunity.
Assadi et al. [5]	Vaccination is important for children	Saving lives vs. preventing adverse events Risk-benefit weighing	Implementing the program in a scientific manner is important, considering the benefits to children and community.
Carrion and Bramstedt [10]	Pediatric organ transplantation. Vaccinations are a tool of organ stewardship	Autonomy Beneficence Paternalism Waiting list mortality Injustice by refusing organ transplantation in unvaccinated children	Vaccine refusals are untenable in the current situation. Encourage vaccination in live donation.
Iserson [11]	Voluntarily unvaccinated individuals pose a threat to children, older adult, and immunocompromised people	Injustice Unfulfillment of social obligations Lower priority for care	Giving lower priority to the voluntarily unvaccinated for admission and for the use of other healthcare resources can be considered ethical as they increase the chance that the COVID-19 virus will mutate and spread, endangering the entire population.
Kraaijeveld et al. [7]	Mandating COVID-19 vaccine for children is unethical	Coercion Paternalism Altruism	When long-term vaccine safety profile for children is unknown and children are not seriously ill nor a major transmitter of the disease, COVID-19 vaccination of healthy children is ethically unjustified.
MacDonald [12]	Coercive Vaccine mandates	Political needs Anti-vaccine protest Public polarization	A need of a better understanding of the political and functional needs of vaccine mandates. A need of better knowledge about the short and long-term outcomes of vaccines.
Mohan et al. [8]	Parents’ hesitance towards COVID-19 vaccination	Complementary vaccine (Ayurveda/naturopathy) is better. Issues trusting vaccine. Lack of information about the future effects of vaccine.	A need to create awareness and acceptance toward the COVID-19 vaccine for children.
Reiss and Caplan [4]	Appropriateness of mandate, legal, practical, and political considerations	Autonomy Beneficence Utilitarianism Justice Non-maleficence	As long as the risk is low, it can be considered ethical to mandate the vaccine.
Ross and Opel [13]	Vaccine mandates in pediatric solid organ transplantation	Graft rejection Safety of the transplantation team Immunosuppressant	Incurrence of harm to unvaccinated children by being unlisted for transplantation.
Savulescu [14]	Mandatory vaccination	Coercion Not a major threat to children Unknown vaccine safety and effectiveness Balancing self-interest with duty to others	Mandating a vaccine on children depends on the nature of the disease, its severity, spread, and the effectiveness of the vaccine itself.
Scendoni et al. [15]	Informed consent	Medical-parent-child- triple therapeutic alliance Parental consent Children’s degree of discernment	The active participation of minors in healthcare decision making is not allowed. The healthcare system should consider empathizing on the minors’ thought and shared solutions.
Wightman et al. [3]	COVID-19 vaccine mandate for transplantation	Best use of organs Transplant gatekeeping Optimize the patients’ chance of survival. Coercive	The undemonstrated effect of SARS-CoV-2 infection in an unvaccinated child due to the impact of immunosuppression vs. the demonstrated effect of the survival, quality of life, and developmental benefits of kidney transplant over dialysis in children. The harm of denial of a transplant is significant.
Williamson [16]	Mandating COVID-19 vaccine	Conflict between health ethics and clinical ethics Individual freedom Trust	The ethical disruption associated with mandating vaccines are to be carefully handled to sustain confidence in vaccination.

**Table 3:** The main themes that emerged from the included studies.

Main Themes (Ethical)	Sub-Themes (Ethical)	Remarks
Mandating COVID-19 Vaccination in children is important	Altruism [7]	The safety of others needs to be considered.
	Political needs [12]	Might result in public polarization against vaccine.
Voluntary un-vaccination	Injustice to others [11]	Threats of disease transmission to younger children, immunocompromised adults, and transplantation team.
	Negligence of care [10,11]	
	Negligence of social obligation [11]	
Ethical challenges in mandating COVID-19 vaccination in children	Coercion [3,7,9,12,14,16]	Coercion is unethical and against the principle of autonomy.
	Autonomy [4,9,10,16]	Parents’ hesitation towards vaccine and unknown long tern side effects results in vaccine refusal.
	Beneficence [4,9,10,14]	Vaccine saves life and pauses the global transmission.
	Utilitarianism [4]	
	Paternalism [7,10,15]	
	Injustice [4,10]	
	Non-maleficence [4]	Vaccines cause certain side effects.
	Risk-benefit ratio [5]	
	Unknown long-term risk [8,14,16]	
Ethical challenges in transplantation	Safety [3,10,17]	Safety of the recipient and the transplantation team from infection.
	Justice [3,10,17]	Vaccines are important but being unlisted for transplantation is injustice to the child.
	Best use of organs [3,10]	Possibility of infection post transplantation due to immunosuppressant.

## Data Availability

All reviewed papers are available in PubMed and WHO COVID-19 Research Database.

## References

[1] Du Z.C., Zhang J., Li X.J., Zhang Z.T., Bai K.S., Wang Z.M., Xu Y., Bai X.W., Sun B. (2022). Impact of COVID-19 pandemic on acute pancreatitis presentations, management, and in-hospital outcomes: A single-center, retrospective observational study from the northeast of China. Ther. Adv. Gastroenterol..

[2] Yu Z., Liang J., Guo L., Jiang L., Wang J.Y., Ke M., Shen L., Zhou N., Liu X. (2022). Psychosocial Intervention on the Dual-Process Model for a Group of COVID-19 Bereaved Individuals in Wuhan: A Pilot Study. Omega.

[3] Wightman A., Goldberg A., Diekema D. (2022). Considering a COVID-19 vaccine mandate for pediatric kidney transplant candidates. Pediatr. Nephrol..

[4] Reiss D.R., Caplan A.L. (2020). Considerations in mandating a new COVID-19 vaccine in the USA for children and adults. J. Law Biosci..

[5] Assadi M., Kiani M., Shamsi Gooshki E., Aryanian Z., Afshar Z.M., Hatami P. (2022). COVID-19 vaccination in children as a global dilemma through an ethical lens: A retrospective review. Health Sci. Rep..

[6] Meo S.A., Bukhari I.A., Akram J., Meo A.S., Klonoff D.C. (2021). COVID-19 vaccines: Comparison of biological, pharmacological characteristics and adverse effects of Pfizer/BioNTech and Moderna Vaccines. Eur. Rev. Med. Pharmacol. Sci..

[7] Kraaijeveld S.R., Gur-Arie R., Jamrozik E. (2022). Against COVID-19 vaccination of healthy children. Bioethics.

[8] Mohan R., Pandey V., Kumar A., Gangadevi P., Goel A.D., Joseph J., Kurien N. (2022). Acceptance and Attitude of Parents Regarding COVID-19 Vaccine for Children: A Cross-Sectional Study. Cureus.

[9] Archard D., Brierley J., Cave E. (2021). Compulsory Childhood Vaccination: Human Rights, Solidarity, and Best Interests. Med. Law Rev..

[10] Carrion L., Bramstedt K.A. (2023). Exploring the ethical complexity of pediatric organ transplant candidates and COVID-19 vaccination: Tensions between autonomy and beneficence, children and parents. Pediatr. Transplant..

[11] Iserson K.V. (2022). Ethics, Personal Responsibility and the Pandemic: A New Triage Paradigm. J. Emerg. Med..

[12] MacDonald N.E., Dubé È., Comeau J. (2022). Vaccines, Politics and Mandates: Can We See the Forest for the Trees? Comment on “Convergence on Coercion: Functional and Political Pressures as Drivers of Global Childhood Vaccine Mandates”. Int. J. Health Policy Manag..

[13] Ross L.F., Opel D.J. (2022). The case against COVID-19 vaccine mandates in pediatric solid organ transplantation. Pediatr. Transplant..

[14] Savulescu J., Giubilini A., Danchin M. (2021). Global Ethical Considerations Regarding Mandatory Vaccination in Children. J. Pediatr..

[15] Scendoni R., Cannovo N., Fedeli P., Cingolani M. (2021). Legal and ethical issues around COVID-19 vaccination consent in Italian children from 12 years of age. J. Leg. Ethical Regul. Issues.

[16] Williamson L. (2021). The ethical impact of mandating childhood vaccination: The importance of the clinical encounter. Clin. Ethics.

[17] Lazarus J.V., Abdool Karim S.S., van Selm L., Doran J., Batista C., Ben Amor Y., Hellard M., Kim B., Kopka C.J., Yadav P. (2022). COVID-19 vaccine wastage in the midst of vaccine inequity: Causes, types and practical steps. BMJ Glob. Health.

[18] Lahariya C. (2008). Mandatory vaccination: Is it the future reality?. Singap. Med. J..

[19] Kata A. (2010). A postmodern Pandora’s box: Anti-vaccination misinformation on the Internet. Vaccine.

[20] Hussain A., Ali S., Ahmed M., Hussain S. (2018). The Anti-vaccination Movement: A Regression in Modern Medicine. Cureus.

[21] Omer S.B., Betsch C., Leask J. (2019). Mandate vaccination with care. Nature.

[22] Patone M., Mei X.W., Handunnetthi L., Dixon S., Zaccardi F., Shankar-Hari M., Watkinson P., Khunti K., Harnden A., Coupland C.A.C. (2022). Risk of Myocarditis After Sequential Doses of COVID-19 Vaccine and SARS-CoV-2 Infection by Age and Sex. Circulation.

[23] Bozkurt B., Kamat I., Hotez P.J. (2021). Myocarditis With COVID-19 mRNA Vaccines. Circulation.

[24] Williams J.T.B., Rice J.D., O’Leary S.T. (2021). Associations between religion, religiosity, and parental vaccine hesitancy. Vaccine X.

[25] Wester C.T., Scheel-Hincke L.L., Bovil T., Andersen-Ranberg K., Ahrenfeldt L.J., Hvidt N.C. (2022). Prayer frequency and COVID-19 vaccine hesitancy among older adults in Europe. Vaccine.

[26] Ali M., Ahmed S., Bonna A.S., Sarkar A.S., Islam M.A., Urmi T.A., Proma T.S. (2022). Parental coronavirus disease vaccine hesitancy for children in Bangladesh: A cross-sectional study. F1000Research.

[27] Aubrey L., Ishak A., Dutta S., Rajesh E., Suvvari T.K., Mukherjee D. (2022). COVID-19 vaccine wastage in Canada, a reason for concern?. Can. J. Public Health.

[28] Zheng Z., Lu Y., Wang M., Luo Y., Wan P., Zhou T., Feng M., Zhu J., Wu J., Ji H. (2023). Low COVID-19 vaccine coverage and guardian acceptance among pediatric transplant recipients. J. Med. Virol..

[29] Young Transplant Recipients Have Better COVID-19 Vaccine Response than Adult Counterparts. https://www.newswise.com/coronavirus/young-transplant-recipients-have-better-COVID-19-vaccine-response-than-adult-counterparts/?article_id=758266.

[30] Shah S., Malik F., Senturia K.D., Lind C., Chalmers K., Yi-Frazier J., Pihoker C., Wright D. (2020). Ethically incentivising healthy behaviours: Views of parents and adolescents with type 1 diabetes. J. Med. Ethics.

[31] Jecker N.S. (2021). Cash incentives, ethics, and COVID-19 vaccination. Science.

[32] Hogan C.M., Waselewski M.E., Szachta P., Wolff C., Amaro X., Chang T. (2022). Perceptions of COVID-19 Vaccine Incentives Among Adolescents and Young Adults. JAMA Netw. Open.

[33] Campos-Mercade P., Meier A.N., Schneider F.H., Meier S., Pope D., Wengström E. (2021). Monetary incentives increase COVID-19 vaccinations. Science.

[34] Sprengholz P., Henkel L., Betsch C. (2022). Payments and freedoms: Effects of monetary and legal incentives on COVID-19 vaccination intentions in Germany. PLoS ONE.

[35] Gong J.D., Barnboym E., O’Mara M., Gurevich N., Mattar M., Anthony D.D., Singer N.G., Perzynski A.T. (2023). Financial Incentives Are Associated with Lower Likelihood of COVID-19 Vaccination in Northeast Ohio. J. Am. Board. Fam. Med..

[36] Shmueli L. (2023). Parents’ intention to vaccinate their 5- to 11-year-old children with the COVID-19 vaccine: Rates, predictors and the role of incentives. BMC Public Health.

[37] Moola S., Gudi N., Nambiar D., Dumka N., Ahmed T., Sonawane I.R., Kotwal A. (2021). A rapid review of evidence on the determinants of and strategies for COVID-19 vaccine acceptance in low- and middle-income countries. J. Glob. Health.

[38] Solís Arce J.S., Warren S.S., Meriggi N.F., Scacco A., McMurry N., Voors M., Syunyaev G., Malik A.A., Aboutajdine S., Adeojo O. (2021). COVID-19 vaccine acceptance and hesitancy in low- and middle-income countries. Nat. Med..

[39] Lazarus J.V., Wyka K., White T.M., Picchio C.A., Gostin L.O., Larson H.J., Rabin K., Ratzan S.C., Kamarulzaman A., El-Mohandes A. (2023). A survey of COVID-19 vaccine acceptance across 23 countries in 2022. Nat. Med..

[40] El Kheir-Mataria W.A., Saleh B.M., El-Fawal H., Chun S. (2023). COVID-19 vaccine hesitancy among parents in Low- and Middle-Income Countries: A meta-analysis. Front. Public Health.

[41] Alimoradi Z., Lin C.-Y., Pakpour A.H. (2023). Worldwide Estimation of Parental Acceptance of COVID-19 Vaccine for Their Children: A Systematic Review and Meta-Analysis. Vaccines.

[42] Lee M., Seo S., Choi S., Park J.H., Kim S., Choe Y.J., Choi E.H., Kwon G.Y., Shin J.Y., Choi S.Y. (2022). Parental Acceptance of COVID-19 Vaccination for Children and Its Association with Information Sufficiency and Credibility in South Korea. JAMA Netw. Open.

[43] Kaufman J., Ryan R., Walsh L., Horey D., Leask J., Robinson P., Hill S. (2018). Face-to-face interventions for informing or educating parents about early childhood vaccination. Cochrane Database Syst. Rev..

[44] Cui Z., Liu L., Li D., Wu S.J., Zhai X. (2022). Safety Messaging Boosts Parental Vaccination Intention for Children Ages 5–11. Vaccines.

[45] Fisher C.B., Bragard E., Jaber R., Gray A. (2022). COVID-19 Vaccine Hesitancy among Parents of Children under Five Years in the United States. Vaccines.

[46] Giannakou K., Kyprianidou M., Heraclides A. (2022). Attitudes and Determinants of Mandatory Vaccination against COVID-19 among the General Population of Cyprus: A Nationwide Cross-Sectional Study. Vaccines.

[47] Askarian M., Semenov A., Llopis F., Rubulotta F., Dragovac G., Pshenichnaya N., Assadian O., Ruch Y., Shayan Z., Padilla Fortunatti C. (2022). The COVID-19 vaccination acceptance/hesitancy rate and its determinants among healthcare workers of 91 Countries: A multicenter cross-sectional study. EXCLI J..

[48] Horiuchi S., Sakamoto H., Abe S.K., Shinohara R., Kushima M., Otawa S., Yui H., Akiyama Y., Ooka T., Kojima R. (2021). Factors of parental COVID-19 vaccine hesitancy: A cross sectional study in Japan. PLoS ONE.

[49] Silwal S., Dhimal M., Bista B., Acharya A., Parajuli K., Pant S., Poudyal A., Ghimire A., Gyanwali P. (2022). Compliance with Social Distancing, Face Mask and Sanitizer Use Measures against COVID-19 in Kathmandu Valley. J. Nepal Health Res. Counc..

